# Cardiac Magnetic Resonance Imaging (MRI) Findings in Arrhythmogenic Right Ventricular Dysplasia (ARVD) Compared with Echocardiography

**DOI:** 10.3390/medsci6030080

**Published:** 2018-09-19

**Authors:** Marzie Motevali, Zainab Siahi, Ali Mohammadzadeh, Akbar Sangi

**Affiliations:** 1 Department of Radiology and Cardiovascular Imaging, Shahid Rajaie Cardiovascular Medical and Research Center, Iran University of Medical Sciences, Tehran 1995614331, Iran; marzieh57@gmail.com (M.M.); ali.mohammad@gmail.com (A.M.); 2 Department of Radiology, Tehran University of Medical Sciences, Tehran 1995614331, Iran; Akbar.sangi@gmail.com

**Keywords:** magnetic resonance imaging, arrhythmogenic, right ventricular, echocardiography

## Abstract

Arrhythmogenic right ventricular dysplasia (ARVD) is an abnormality in the right side of the heart that may lead to sudden death. The study aims to compare cardiac MRI (magnetic resonance imaging findings) with echocardiography in patients with ARVD. For the cross-sectional study, patients with ARVD that were diagnosed using Task Force criteria were included, and their cardiac MRI findings were evaluated. Additionally, the right ventricle was divided into three levels—basal, middle, and apical—and each of them was also subdivided into three secondary segments. Gadolinium enhancement was evaluated in each segment. Overall, 39 patients were studied. Thirty-one patients (81%) were men. The average age of female and male patients was 37.8 ± 4.6 and 32.48 ± 5.8, respectively. The average ejection fraction found was 43 ± 9.4 and 42.8 ± 8.5% by MRI and echocardiography, respectively. Additionally, 46 and 35.8% of the patients had hypokinesia in the right ventricle, found based on MRI and echocardiography, respectively. The right ventricular aneurysm was found in 20.5 and 5.1% of patients based on MRI and echocardiography, respectively. The cardiac MRI managed to diagnose some cases which echocardiography was not able to detect. Thus, MRI plays an important role in presenting diagnostic data for the management of patients with ARVD and also making the diagnosis in suspicious patients definitive.

## 1. Introduction

Arrhythmogenic right ventricular dysplasia (ARVD) is a type of cardiomyopathy that primarily affects the heart muscle function and is frequently familial but also observed sporadically [1]. Arrhythmogenic right ventricular dysplasia accounts for 5% of sudden death in adults. It is seen particularly in young adults, and 80% of the cases diagnosed are in individuals under the age of 40 years. The prevalence of ARVD is not exactly known, however, it has been estimated at 1:5000 in America [2]. In ARVD, the cells of the heart muscle, mainly right ventricle, are replaced by fat and fibrous tissue. Historically, two common histopathologic patterns have been described [3]. The first one is Fibrolipomatosis Type 1 in which more fat tissue along with a small amount of fibrous tissue enclosed by healthy myocytes is observed. In this type, wall thickness increases and right ventricular pseudohypertrophy occurs. In Fibrolipomatosis Type 2, myocytes are extensively replaced by fat and fibrous tissue, which leads to saccular aneurysm and ventricular septal aneurysm [4].

According to cellular and molecular studies, cellular desmosome dysfunction mainly causes ARVD. Desmosomes are membrane proteins that cause cells to adhere together, and disorder in the function of these proteins affects muscle and skin cells most of all [5]. Desmosomes also contain various intracellular proteins. In addition to cell-to-cell adherence, these proteins have a role in cell communication and tissue differentiation. Disorder in each of these proteins, dependent on the intensity, can provide the condition for the occurrence of ARVD. The disease can also appear as the effect of an inflammatory disease or as a type of myocarditis. Nevertheless, the role of the cell death process [6], viral infections [7,8], and genetic predisposition to ARVD is still unknown. ARVD usually has no symptoms and may even cause sudden death. However, arrhythmia and conduction disorders such as a left bundle branch block (LBBB) and right bundle branch block (RBBB) are common clinical manifestations of ARVD which are usually diagnosable through electrocardiography [9].

Echocardiography is a key method for diagnosing ARVD which facilitates the recognition of right ventricular morphological abnormalities and other associated pathologies, including ventricular septal disorders like thinning and trabeculation. Although, given that an abnormality in myocardial function has no effects on the whole function of right ventricle at the initial stages, diagnosis of ARVD by echocardiography is difficult at this stage [10].

Based on study reports, by detecting microaneurysm, wall bulging, and fibrous tissue, and calculating the volume of right and left ventricles and the ejection fraction and comparing them with echocardiography results, magnetic resonance imaging (MRI) can have an efficient role in diagnosing ARVD and related pathologies. However, detection of intramyocardial fat in the right ventricle by MRI is unreliable, and the contact of pericardial fat with a thin right ventricle wall leads to misdiagnosis of fat tissue infiltration in the heart [11].

Limited studies have compared diagnostic results of MRI and echocardiography in ARVD patients that are candidates for ablation therapy [12,13]. Therefore, the present study aims to investigate cardiac MRI findings in patients with ARVD compared to echocardiography and, thus, present proper indexes for a better diagnosis of the disease.

## 2. Materials and Methods

In this cross-sectional study, 39 patients with ARVD that were diagnosed based on Task Force criteria [11] and were candidates for ablation at the Tehran Shahid Rajaei Heart Centre during 2017 to 2018 were investigated. This study was approved by the Iran University of Medical Sciences ethical committee (ethical code: 11-1395).

### 2.1. Demographics and Clinical Status

Patients’ demographic characteristics such as age, sex, and body mass index (BMI) and their echocardiography findings were recorded in each patient questionnaire. Disease, surgery, and medication history, as well as family history, were asked from the patients and then recorded.

### 2.2. Measure

First, the results of patients’ echocardiography (including the ejection fraction, presence of hypokinesia and/or aneurysm in right ventricle, average right ventricle outflow tract or RVOT, and the presence of fibrous tissue and fat deposits in heart) were recorded. Then, the cardiac MRI was carried out for the patients. To explore the images more precisely, the right ventricle was divided into three levels, namely basal, middle, and apical. Each of them were also subdivided into three secondary segments (i.e., superior, midwall, and inferior). Gadolinium enhancement in each segment was evaluated based on 0 and 1 scoring, and a score between 0 and 9 was given to each patient.

Contrast-to-noise ratio (CNR) in the right ventricle; the volume of the right ventricle; and functional abnormalities such as regional wall motion abnormalities (RWMAs), right ventricular dilatation and systolic-diastolic dysfunction in right ventricle, right ventricular aneurysm and segmental hypokinesia, intramyocardial fat infiltration, focal wall thinness, wall hypertrophy, trabecular hypertrophy, and RVOT hypertrophy were also evaluated for each patient.

### 2.3. Statistic Analysis

Finally, obtained results were analyzed using SPSS (Version 22). Continuous data were reported in the form of mean and standard deviation (Mean ± S.D.) and discrete data in the form of frequency (percent/number). To analyze the data, Mann-Whitney U and Fisher tests were used. To find the relationship between the variables, regression, Pearson tests and, if needed, the kappa (κ) agreement test were applied. Having a kappa coefficient of less than 0.2 was considered as a poor relationship, 0.4 to 0.6 as moderate, and over 0.6 as good. The *p*-value of the test was 0.05.

## 3. Results

Overall, 39 patients were studied of whom 31 (80%) were men and 8 (20%) women. The total average ages of female and male patients were calculated to be 37.8 ± 4.6 and 32.48 ± 5.8, respectively.

Generally, there were significant MRI changes in about a third of the patients diagnosed with ARVD. Using MRI, the average ejection fraction (EF) was calculated to be 43 ± 3.4% (between 23 and 59%). Using echocardiography, this variable was instead calculated to be 42.8 ± 8.5% (between 30 and 55%). Statistical analysis and kappa agreement showed poor consistency and significant difference between average EF measured by the two techniques (*p* = 0.01) (Kappa = 0.2).

The number of patients with right ventricular hypokinesia diagnosed by MRI and echocardiography were 18 (46%) and 14 (35.8%), respectively. Diagnosis of right ventricular hypokinesia by MRI and echocardiography had a nearly similar accuracy (*p* = 0.1) (Kappa = 0.7).

The number of patients diagnosed with right ventricular aneurysm by MRI and echocardiography were eight (20.5%) and two (5.1%) respectively. Statistical analysis and the kappa agreement test showed that there was no consistency between diagnoses by MRI and echocardiography. In other words, the diagnosis of right ventricular aneurysm by MRI was superior to echocardiography (*p* = 0.05) (Kappa = 0.00).

One patient with fibrous tissue was diagnosed using MRI (2.56%), while no cases of fibrous tissue were found using echocardiography. Statistical analysis indicated the similar ability of MRI and Echocardiography to diagnose fibrous tissue (*p* = 0.08) (Kappa = 0.7).

Furthermore, MRI showed fat tissue deposit in three patients, while echocardiography showed no cases of fat tissue deposit. Statistical analysis also indicated the inconsistency between MRI and echocardiography (*p* = 0.05) (Kappa = 0.00).

An average right ventricular outflow tract (RVOT) measured by MRI was 31.17 ± 102 mm (between 21 and 50 mm). Table 1 shows parameters of right ventricular dilatation measured by MRI.

**Figure 1:**
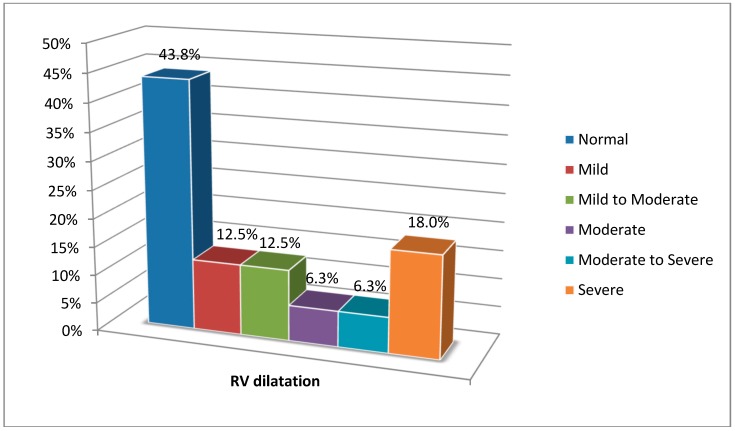
Rating of right ventricular dilatation severity in the patients based on echocardiography.

The severity of right ventricular dilatation in the patients was rated based on the echocardiography technique, and this has been depicted in detail in Figure 1.

Additionally, the severity of systolic dysfunction evaluated by echocardiography has also been presented in Table 2.

Magnetic resonance imaging detected enlargement of the right ventricle in eight patients (33%) and right ventricular hypertrophy in 8.3% of patients. Echocardiography was also able to detect right ventricular hypertrophy in 4.2% of patients (*p* = 0.03).

## 4. Discussion

Arrhythmogenic right ventricular dysplasia is a disorder with diagnostic features and aspects which are not completely known yet. Therefore, discovering proper diagnostic methods is still a controversial issue [12]. However, MRI can be used as a first-line diagnostic method to identify some specific features of the disease [13,14]. According to studies, MRI is considered as the golden standard for measuring the volume of cardiac masses and, due to high accuracy, is a better option compared to other imaging methods in cardiology [15,16]. Nevertheless, the inability to complete the determining Task Force Criteria for diagnosis of the disease is one of the main disadvantages of MRI that, in some cases, can cause misdiagnosis. Furthermore, there are some common false beliefs, including that some radiologists believe that fat tissue in the myocardium is a definitive sign of ARVC and that MRI is the best tool for diagnosing fat tissue deposits in myocardium. However, a high signal in MRI should not be interpreted as the presence of the disease and observing it in any condition does not show the presence of fat tissue deposit. Various amounts of fat tissue are even observed in the right ventricle of patients without ARVC and every fat signal does not show ARVC [16,17]. According to study of Bomma et al. [18], in some cases, observing fat signal can be misleading in diagnosing ARVC. Thus, a fat signal is more helpful to determine the clinical history of the patient.

The presence and severity level of motion disorder and/or aneurysm in the right ventricle can be only confirmed after precise investigation. Proficient radiologists’ experience in interpreting disorder in the right ventricle wall contributes considerably to detecting its location.

Based on research, the left ventricular function in patients with ARVC/D may significantly decrease more than right ventricle. As a result, in some cases, patients with ARVC/D do not lie in the diagnostic criteria for this disease because they rely more on reduction in right ventricular function [19,20].

Difference between ARVD diagnostic criteria in MRI and echocardiography poses a question as to what extent obtained results are reliable.

Based on a study using MRI on 41 patients suspected of having ARVD, the disease was definitively confirmed only in 60% of them [11]. Possible reasons for different interpretations of imaging by other techniques may be that this disease is an uncommon disorder and requires special diagnostic protocols for desired evaluation. In addition, in most centers for diagnostic criteria evaluation, quantitative diagnostic criteria are applied as much as possible. For example, in MRI Centers, short axis images may not be prepared, which leads to misdiagnosis and mismeasurement of right heart dimensions in 20% of cases. Moreover, MRI interpretation may be carried out by incompetent individuals with limited experience in differentiating this disease from others, since detection of pathologic changes in RV natural structure in ARVD, especially at the initial stages, is difficult. Also, according to some studies, there may be a high error level in differentiating natural motion from RV pathology using MRI, especially near moderator band [20]. Furthermore, considerable diagnostic errors have been reported regarding fat deposits in RV, as well as RV wall thinness [21]. In non-pathologic heart conditions, there is an amount of fat tissue deposit in RV wall, especially in the anterolateral and apical regions, and these findings should be considered when results obtained from the patients suspected of having ARVD are interpreted. On the other hand, in some cases, viral myocarditis can imitate ARVD clinical manifestations and/or predispose patients with ARVD to viral myocarditis that translates into a reduction in RV function and exacerbation of the disease [22].

Previous studies on ischemic and nonischemic cardiomyopathy indicate a delay in nonspecific tissue enhancement, but it can also result from an increase in secondary gadolinium distribution volume due to intratissue space widening, myocardial fibrous tissue, and/or the presence of inflammation. Delay in enhancement occurs more in RVOT and anterobasal regions because of more fat or fibrous tissue deposit in the two regions. Wall motion abnormality also occurs more in these areas, which indicates cardiac cell dysfunction in the same region. These observations increasingly support the theory that the presence of fibrous diffused regions and, thus, the localization of gadolinium in these areas are the main mechanism for delay in enhancement in patients with ARVD [17,23].

## 5. Conclusions

Magnetic resonance imaging (MRI) is an important method for diagnosing ARVC; however, diagnosis of the disease by MRI has abundant diagnostic precision. This disease is rare, and most centers lack remarkable experience in evaluating ARVC diagnostic criteria. On the other hand, technical problems and the non-existence of standard MRI protocols for ARVC diagnosis indicate the necessity of a complete evaluation of these patients through Task Force Criteria. Generally, MRI plays an important role in presenting diagnostic data for the management of patients with ARVC and also making a definitive diagnosis in patients suspected of having the disease.

## Figures and Tables

**Table 1:** Parameters related to right ventricular dilatation measured by MRI.

Parameter	Mean ± S.D.
RVEDV ^1^	190.6 ± 88
RVESV ^2^	116.52 ± 68.9
SV ^3^	75.3 ± 31.2
RVEDVI ^4^	103.6 ± 49.4
RVESVI ^5^	60.9 ± 41

RVEDV ^1^: right ventricular end diastolic volume; RVESV ^2^: right ventricular end systolic volume; SV ^3^: systolic volume; RVEDVI ^4^: right ventricular end diastolic volume index; RVESVI ^5^: right ventricular end systolic volume index.

**Table 2:** Rating of systolic dysfunction severity in the patients measured by echocardiography.

Systolic Function	Frequency (%)
Normal	21%
Mild	21%
Mild to moderate	42%
Moderate to severe	5.3%
Severe	10.5%
